# Integration of Well‐Being Therapy and Positive Psychotherapy: A Response to Fava and Guidi's (2021) Commentary on Radstaak et al. (2020)

**DOI:** 10.1002/jts.22671

**Published:** 2021-03-14

**Authors:** Mirjam Radstaak, Laura Hüning, Ernst T Bohlmeijer

**Affiliations:** ^1^ Department of Psychology Health and Technology University of Twente Enschede The Netherlands; ^2^ Mediant Community Mental Health Center Enschede The Netherlands

## Abstract

The results of our study on the effects of well‐being therapy (WBT) compared with a treatment‐as‐usual (TAU) control condition among individuals with residual symptoms of posttraumatic stress disorder (PTSD) were recently published in the *Journal of Traumatic Stress* (Radstaak et al., 2020). In a subsequent commentary, Fava and Guidi (2020) raised several conceptual and methodological issues that they asserted potentially limited the interpretation of the results. In this response, we aim to clarify these issues, thus contributing to the optimal interpretation of the findings.

In 2020, the results of our study on the effects of well‐being therapy (WBT) compared with treatment as usual (TAU) among individuals with residual symptoms of posttraumatic stress disorder (PTSD) were published in this journal (Radstaak et al., 2020). In a subsequent commentary, Fava and Guidi (2021) asserted that the WBT intervention offered to participants with residual PTSD symptoms in our study cannot be defined as such. We termed the intervention WBT given that some of the main components of WBT as developed by Fava (1999) were applied. For example, participants were encouraged to use a diary to identify episodes of well‐being, asked to identify thoughts leading to premature interruption of well‐being, asked to learn and apply skills to challenge and replace maladaptive cognitions, introduced to interventions that aimed to transform impaired levels of psychological well‐being to optimal levels, and invited to do several exercises at home each week. In addition, we conducted an assessment of the six levels of psychological well‐being.

However, we made one important adaptation in our study. One of the aims of WBT is to elicit psychological well‐being and optimal experiences (Fava, 1999; Fava & Guidi, 2021); thus, we integrated positive psychotherapy interventions into the six sessions of WBT to enhance those feelings and experiences. In the first session, the importance of positive emotions and exercises to promote positive emotions were introduced, as positive emotions have been shown to be related to “human flourishing” (Fredrickson, 2004; Fredrickson & Losada, 2005). Given that compassion also elicits psychological well‐being, the third session of our intervention focused on compassion and exercises to promote compassion (Neff, 2004; Zessin et al., 2015). In the fourth session, participants learned about the construct of posttraumatic growth (PTG) and were presented with assignments designed to increase PTG. These assignments were added because growth following adversity has been shown to be associated with psychological well‐being (Joseph & Linley, 2005; Durkin & Joseph, 2009). Integrating these interventions allowed our intervention to promote psychological well‐being in a relatively brief period. In sum, due to its brevity and the adaptations that were made, the therapy described in Radstaak et al. (2020) can best be characterized as a new, integrative intervention that combines components of WBT positive psychotherapy.

In their Commentary, Fava and Guidi (2021) pointed out two concerns about the control condition, the first of which was regarding the operationalization of the control condition. The authors argued that what was called TAU could be better defined as an active, psychoeducational treatment. We agree with Fava and Guidi (2021) that defining our control condition as an active, psychoeducational treatment fits the control condition well. An active psychoeducational control condition implies a control condition that maintains. This definition implies a control condition that maintains treatment fidelity by adhering to a clearly defined, manualized therapeutic procedure that is defined by prescriptions rather than proscriptions and allows researchers to control for nonspecific treatment components (Mohr et al., 2009). Nevertheless, we prefer the term TAU because the control condition in our study represented the TAU administered at the institution where the study was conducted.

Fava and Guidi (2021) raise a second concern that due to its focus on the pursuit of goals, the TAU condition had more similarities to WBT than to the experimental condition. Successful goal attainment can indeed increase psychological well‐being, but holding goals does not necessarily lead to goal attainment and can even undermine well‐being (Boudreaux & Ozer, 2013). Nonetheless, participants in the TAU condition (a) did not identify episodes of well‐being, (b) did not identify thoughts and behaviors that interrupt well‐being, (c) were not monitored for specific impairments of well‐being, and (d) were not encouraged to attain an optimally balanced state of functioning. Thus, the assertion that the TAU differed from the original manualized WBT less than the experimental group does not seem to be warranted.

Fava and Guidi (2021) called for an “individualized therapeutic WBT plan” (p. 2) based on macroanalysis and staging and argued that the final sample of our study “was highly heterogeneous with regard to the patient's treatment history” (p. 2), which makes “any conclusion difficult to draw” (p. 2) regarding whether WBT was more effective than TAU (Radstaak et al., 2020). Indeed, we did not account for individuals’ treatment history and past remission; however, participants were included in the sample only when they no longer met the diagnostic criteria for PTSD. Thus, all of our participants completed an effective treatment, which reduced the heterogeneity of our sample and placed participants in the residual phase of a disorder (Fava et al., 2011). We agree fully with Fava and Guidi's (2021) assertion that it is important to individualize WBT treatment. In fact, we stated in our article that “personalized interventions for health should include clinically relevant individual characteristics, such as differences in well‐being” (Radstaak et al., 2020, pp. 819–820). Researchers have emphasized the importance of the measurement of clinical issues that are not part of customary clinical taxonomy, such as well‐being (Fava & Guidi, 2020; Fava et al., 2011). To our knowledge, our study was the first to provide empirical data on well‐being.

In our article, we presented the results of a pragmatic, randomized controlled trial conducted to assess the impact of a brief intervention combining WBT and positive psychotherapy for patients with residual PTSD symptoms compared to a TAU control condition (Radstaak et al., 2020). Despite some apparent limitations, the study results suggest that a brief intervention that aims to promote psychological well‐being may be effective for patients with residual symptoms of PTSD and low levels of well‐being.
